# Chemosensory Proteins Are Associated with Thiamethoxam and Spirotetramat Tolerance in *Aphis gossypii* Glover

**DOI:** 10.3390/ijms23042356

**Published:** 2022-02-21

**Authors:** Hongfei Xu, Kunpeng Yan, Yaping Ding, Yuntong Lv, Jianyi Li, Fengting Yang, Xuewei Chen, Xiwu Gao, Yiou Pan, Qingli Shang

**Affiliations:** 1 College of Plant Science, Jilin University, Changchun 130062, China; xuhf19@mails.jlu.edu.cn (H.X.); yankp8218@mails.jlu.edu.cn (K.Y.); dingyp21@mails.jlu.edu.cn (Y.D.); ytlv20@mails.jlu.edu.cn (Y.L.); jianyi21@mails.jlu.edu.cn (J.L.); yangft20@mails.jlu.edu.cn (F.Y.); 2 School of Agricultural Science, Zhengzhou University, Zhengzhou 450001, China; chen_xw2010@163.com; 3 Department of Entomology, China Agricultural University, Beijing 100193, China; gaoxiw@263.net.cn

**Keywords:** chemosensory protein, thiamethoxam, spirotetramat, *Aphis gossypii*, insecticide resistance

## Abstract

Chemosensory proteins (CSPs) are a class of transporters in arthropods. Deeper research on CSPs showed that CSPs may be involved in some physiological processes beyond chemoreception, such as insect resistance to pesticides. We identified two upregulated *CSPs* in two resistant strains of *Aphis gossypii* Glover. To understand their role in the resistance of aphids to pesticides, we performed the functional verification of *CSP1* and *CSP4* in vivo and in vitro. Results showed that the sensitivity of the thiamethoxam-resistant strain to thiamethoxam increased significantly with the silencing of *CSP1* and *CSP4* by RNAi (RNA interference), and the sensitivity of the spirotetramat-resistant strain to spirotetramat increased significantly with the silencing of *CSP4*. Transgenic *Drosophila melanogaster* expressing *CSPs* exhibited stronger resistance to thiamethoxam, spirotetramat, and alpha-cypermethrin than the control did. In the bioassay of transgenic *Drosophila*, *CSPs* showed different tolerance mechanisms for different pesticides, and the overexpressed *CSPs* may play a role in processes other than resistance to pesticides. In brief, the present results prove that *CSPs* are related to the resistance of cotton aphids to insecticides.

## 1. Introduction

With the extensive use of chemical insecticides in global agriculture and horticulture, pests have developed resistance to various insecticidal modes of action [1]. To ensure that current and future pesticides can continue to play a leading role in resisting pests, effective insecticide resistance management (IRM) is essential [2]. An effective approach is to understand the resistance mechanism and formulate different strategies on the basis of different mechanisms. Four main resistance mechanisms are widely investigated [3,4,5,6]. The first is target-site resistance [5], for example, the KDR mechanism was first reported in several pyrethroid-resistant clones of *Myzus persicae* in 1997 [7]. Studies showed that RyR mutations are associated with high levels of chlorantraniliprole resistance in *Plutella xylostella* (L.) [8,9]. In addition, penetration resistance was described as cuticle thickening and the alteration of cuticle composition [6]. In *Anopheles gambiae*, the hydrocarbons synthesized by two overexpressed cytochrome P450 monooxygenases (P450s), namely, *CYP4G16* and *CYP4G17*, enriched deposition on the epicuticle and significantly reduced the rate of internalization of ^14^C deltamethrin [10]. Several *ABCGs* enriched in mosquito legs were found to be overexpressed in populations of different insecticide-resistant strains [11]. Another mechanism is behavioral resistance, which remains controversial. Some reports state that behavioral resistance is only acquired or based on simple repellency or avoidance [3]. This mechanism was first proposed in 1956 [12], and evidence of genetic changes similar to those for other resistance mechanisms were still lacking until recently, when a report showed that glucose-averse (GA) cockroaches turned the stimulation by ‘sugar baits’ of the glucose receptor into a deterrent effect and then avoided feeding on poisonous bait [13]. The last and most important type is metabolic resistance. This type of resistance is usually achieved by increasing P450s, uridine diphosphate (UDP)-glycosyltransferase (UGTs), glutathione S-transferases (GSTs), and carboxylesterases (CarEs) [14,15,16,17]. The overexpression of *CYP6BG1* may be related to the resistance of *P. xylostella* to chlorantraniliprole [18]. Exposure to multiple insecticides can upregulate the expression of three UGT genes in *P. xylostella* (L.) [15]. In resistant *Bemisia tabaci* strains, the expression level of *GST-d7* was significantly increased compared with that in sensitive strains. The knockdown of *GST-d7* in a resistant strain significantly increased the sensitivity of *B. tabaci* to imidacloprid [19]. The upregulation of CarE leads to increased resistance of cotton aphids to deltamethrin [14]. Previous studies on metabolic resistance have mostly focused on these traditional detoxification enzymes, while the functions of other proteins are often overlooked.

CSPs are small water-soluble peptides with low isoelectric points that are only found in arthropods [20,21]. They have four conserved cysteines (4 Cys proteins) and a strong affinity for small hydrophobic molecules [20]. CSPs respond sensitively to biologically significant chemical signals in the surrounding environment [22]. They are expressed in the whole body rather than only in chemoreception organs. Beyond chemoreception, CSPs also play an important role in many other physiological functions in insects [20,23]. In 2003, the emergence of reports about OS-D/A10 and *Drosophila* immunity provided a new direction for exploring the function of small-molecule proteins such as CSPs [24]. Subsequently, increasing evidence indicated that CSPs might be involved in insect resistance to pesticides. A study reported that sensory appendage protein SAP2 is related to the resistance of *A. gambiae* to pyrethroids [25]. Moreover, with a single short-term exposure to the insecticide abamectin, the expression of twenty *CSPs* increased dramatically in adults of silkworm *Bombyx mori* [26]. The expression of *CSP4* and *CSP8* in *P. xylostella* was significantly upregulated under long-term exposure to different permethrin concentrations [27]. *CSP1* exhibits thiamethoxam-mediated upregulation in *B. tabaci*, and CSP2 and CSP3 show a high binding affinity to α-pentyl-cinnamaldehyde, which is a common chemical in plant oil with toxic effects upon direct contact [28]. The above studies showed that CSPs may be related to insect xenobiotic detoxification, and their mechanisms of action are different from those in the past. At this stage, the function of CSPs in the resistance of *A. gossypii* to insecticides is still unclear.

Thiamethoxam is a neonicotinoid insecticide that is widely used to control *A. gossypii* by binding to nicotinic acetylcholine receptors (nAChRs) [29,30]. Multiple field populations of cotton aphids have developed different resistance levels to thiamethoxam [31]. In addition, the tetramic acid derivative spirotetramat can inhibit insect acetyl-CoA carboxylase (ACC) from decreasing lipid biosynthesis [32,33,34]. This insecticide is outstanding in resisting sucking pests in laboratory assays and field trials [35]. Currently, the field population of *B. tabaci* has a certain level of resistance to spirotetramat [36], but the field population of cotton aphids does not.

To clarify the potential roles of CSPs in the resistance of *A. gossypii* to pesticides, three strains cotton aphids were used in this study, include susceptible (SS) strain, thiamethoxam-resistant (ThR) strain and spirotetramat-resistant (SR) strain. We performed functional analysis of two *CSPs* that are highly expressed in cotton-aphid-resistant strains. Thiamethoxam and spirotetramat were selected as the test pesticides. Upregulated *CSPs* were identified in the transcriptome [37]. RNA interference (RNAi) in aphids and ectopic expression in *Drosophila* were used to verify the capability of *CSPs* in vivo and in vitro. Our research can provide a basis for obtaining a comprehensive understanding of insect resistance mechanisms and xenobiotic detoxification.

## 2. Results

### 2.1. Different Expression Levels of CSP1 and CSP4 in Diverse Strains

Two overexpressed *CSPs* were found by analyzing transcriptome data (Table 1) [37]. The expression levels of *CSP1* in the ThR strain, and *CSP4* in the ThR and SR strains significantly increased compared with those in the SS strain. RT-qPCR results were in accordance with the transcriptome data (Table 1). *CSP1* showed a higher expression level in the ThR strain than that in the SS strain. The expression level of *CSP4* was upregulated to 2.30- and 6.08-fold in the ThR and SR strains, respectively.

### 2.2. RNAi Increases Thiamethoxam and Spirotetramat Toxicity

**Figure 1:**
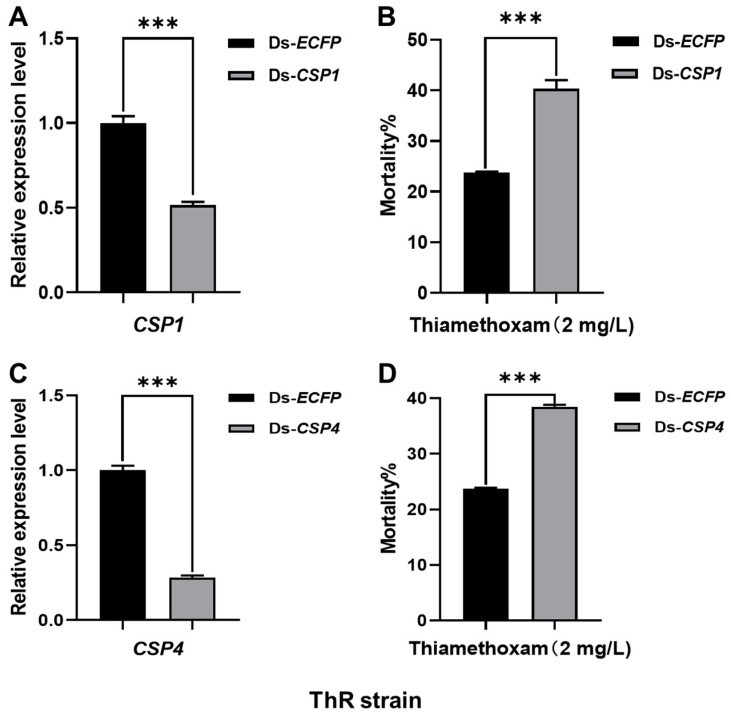
dsRNA-mediated suppression of *CSP1* and *CSP4* transcription and its effect on thiamethoxam toxicity in ThR strain aphids. (**A**) dsRNA-mediated suppression of *CSP1* transcription in adult ThR strain aphids fed an artificial diet containing dsRNA (150 ng/μL). (**B**) Mean mortality ± SE (*n* = 3) of ThR strain cotton aphids after being fed the mixture of thiamethoxam (final concentration: 2 mg/L) and dsRNA-*CSP1* (final concentration: 150 ng/μL) for 48 h, a diet with dsRNA-*ECFP* as control. (**C**) dsRNA-mediated suppression of *CSP4* transcription in adult ThR strain aphids fed an artificial diet containing dsRNA (150 ng/μL). (**D**) Mean mortality ± SE (*n* = 3) of SR strain cotton aphids after being fed the mixture of thiamethoxam (final concentration: 2 mg/L) and dsRNA-*CSP4* (final concentration: 150 ng/μL) for 48 h, a diet with dsRNA-*ECFP* as control. Each treatment included three replicates, and eighty resistant adult aphids were used in each replicate. Error bars indicate 95% confidence intervals (*n* = 3). *** Significant difference by Student’s *t* test (*p* < 0.001).

**Figure 2:**
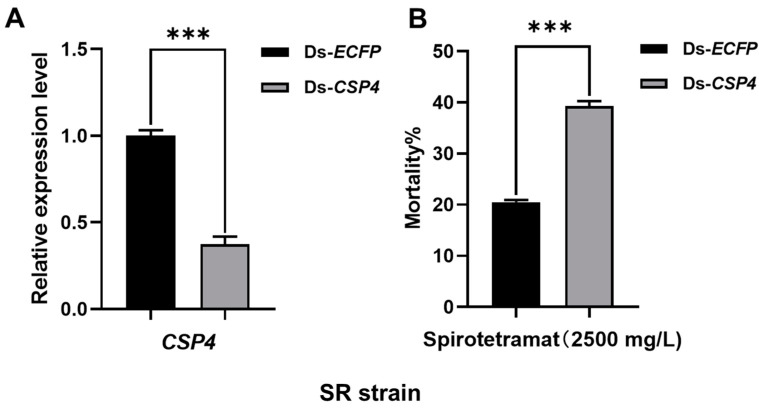
dsRNA-mediated suppression of *CSP4* transcription and its effect on spirotetramat toxicity in adult SR strain aphids. (**A**) dsRNA-mediated suppression of *CSP4* transcription in adult SR strain aphids fed an artificial diet containing dsRNA (150 ng/μL). (**B**) Mean mortality ± SE (*n* = 3) of SR strain cotton aphids after being fed the mixture of spirotetramat (final concentration: 2500 mg/L) and dsRNA-*CSP4* (final concentration: 150 ng/μL) for 48 h, a diet with dsRNA-ECFP as control. Each treatment included three replicates, and eighty resistant adult aphids were used in each replicate. Error bars indicate 95% confidence intervals (*n* = 3). *** Significant difference by Student’s *t* test (*p* < 0.001).

To verify the relationships of the CSPs and resistance to insecticides, RNAi assays were conducted. After feeding the diet with the corresponding dsRNA (150 ng/μL) for 48 h, the expression levels of *CSP1* and *CSP4* were significantly decreased. qPCR results showed that the transcriptional levels of *CSP1* were reduced 0.52-fold (*p* = 0.0004) in the ThR strain (Figure 1A), and those of *CSP4* were reduced 0.28-fold (*p* < 0.0001) in the ThR strain (Figure 1C) and 0.37-fold (*p* = 0.003) in the SR strain (Figure 2A). The silencing of *CSP1* and *CSP4* significantly increased the mortality of ThR strain aphids from 24% in the control to 40% (*p* = 0.0006) and 38% (*p* < 0.0001), respectively, under thiamethoxam treatment (Figure 1B,D). Moreover, with spirotetramat treatment, the mortality of the SR strain aphids under silencing of *CSP4* significantly increased from 21% to 39% (*p* < 0.0001) (Figure 2B).

### 2.3. Ectopic Expression of CSPs Enhanced Drosophila Tolerance

**Figure 3:**
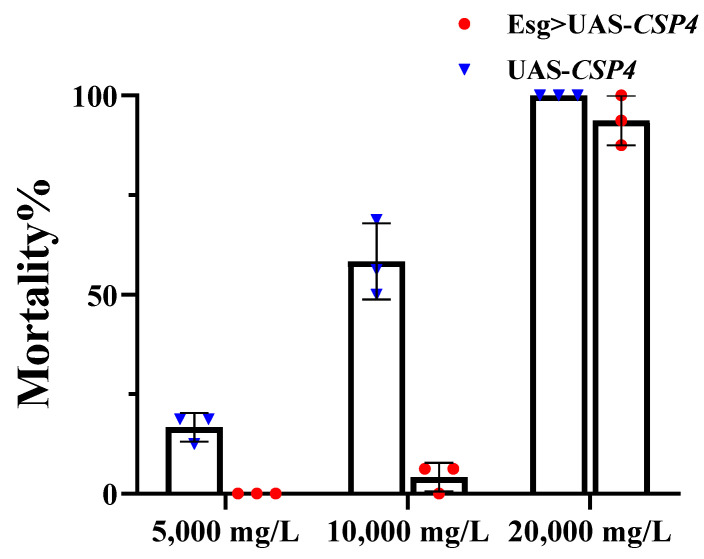
Gastric toxicities of spirotetramat to transgenic *Drosophila melanogaster* with *CSP4*. Mean mortality ± SE (*n* = 3) of Esg > UAS-*CSP* F1 adult offspring after being fed the mixture of spirotetramat (final concentration: 5000, 10,000, 20,000 mg/L) for 168 h. Each treatment included three replicates, and eight pairs of 2-day-old *Drosophila* adults were used in each replicate. Error bars indicate 95% confidence intervals (*n* = 3).

To validate the correlation between CSPs and resistance to thiamethoxam, alpha-cypermethrin, and spirotetramat, *CSP1* and *CSP4* were ectopically expressed in *Drosophila* using the GAL4/UAS system. Act5C/Esg > UAS-*CSP* F1 offspring were confirmed by qPCR in adults with a straight-wing phenotype and the expression of green fluorescent protein in larvae (Figures S1–S3 in Supplementary data). In the bioassays of thiamethoxam, the LD_50_ values of transgenic *Drosophila* expressing *CSP1* and *CSP4* increased by 2.80- and 6.76-fold, respectively, compared with those in the control in terms of contact toxicities (Table 2). The LC_50_ values increased 1.37- and 1.23-fold in terms of gastric toxicities (Table 3). In the bioassays of alpha-cypermethrin, the LD_50_ values of contact toxicities showed that the tolerance ability of flies ectopically overexpressing *CSP1* and *CSP4* increased 1.06- and 5.76-fold (Table 2), and the LC_50_ values of gastric toxicity increased by 3.29- and 7.27-fold (Table 3). Except for flies expressing *CSP1* in the alpha-cypermethrin contact treatment, all variations were significant because of nonoverlapping in 95% confidence limits (95% CL). The gastric toxicities of spirotetramat to *Drosophila* are shown in Figure 3. The mortality of flies expressing *CSP4* was 0%, 4.17%, and 93.75% at 5000, 10,000, and 20,000 mg/L, respectively, and that of the control was 16.67%, 58.33%, and 100%, respectively. Mortality at 5000 and 10,000 mg/L was significantly different (*p* < 0.01) between flies expressing *CSP4* and the control flies. The results of contact toxicities and gastric toxicities showed that overexpressed *CSP1* and *CSP4* can help *Drosophila* endure insecticide exposure.

## 3. Discussion

CSPs are small water-soluble peptides with a hydrophobic binding pocket that can bind small molecules [20,21]. Previous studies on CSPs focused on their ability to perceive biologically significant chemical signals in the surrounding environment [22]. It was not until the report that OS-D/A10 may be related to *Drosophila* immunity appeared in 2003 [24] that researchers realized that CSPs might have functions beyond chemoreception [23]. An increasing number of studies reported on CSPs conferring insect resistance [25,26,27,28].

Gene overexpression overcomes the shortcomings of traditional loss-of-function analysis [38]. Currently, upregulated expression of genes is a common standard for identifying genes related to a certain biological process [27]. Numerous reports showed that CSP expression in insects increases with pesticide exposure [25,26,27,28]. We screened the upregulated *CSPs* in the existing ThR and SR strains of cotton aphids by transcriptome sequencing and fluorescence quantification methods (Table 1). A report on *A. gambiae* found that the expression of *SAP2* was upregulated in insecticide resistant populations [25]. Moreover, the expression levels of *CSP1* and *CSP4* were significantly increased in resistant *A. gossypii* strains. Our results may indicate that *CSP1* and *CSP4* are related to the resistance of cotton aphids to insecticides.

RNAi is one of the commonly used methods for the verification of gene function in vivo [39,40]. For example, RNAi-mediated knockdown of *CSP10* significantly increased the susceptibility of *T. castaneum* to dichlorvos or carbofuran [41]. In addition, the suppression of *CSP4*, *CSP5*, *CSP6,* and *CSP10* in *Rhopalosiphum padi* dramatically elevated imidacloprid toxicity [42]. In our study, with the suppression of *CSP1* and *CSP4*, the susceptibility of ThR and SR strain aphids to thiamethoxam and spirotetramat significantly increased (Figure 1 and Figure 2), which suggests that *CSP1* and *CSP4* contribute to the insecticide resistance in *A. gossypii*.

The GAL4/UAS system was extensively used in gene function verification in recent years [25,40,43]. *D. melanogaster*, as a well-characterized model insect, can be examined with a variety of genetic verification methods. For instance, transgenic expression of *CYP380C6*, *CYP6CY7*, *CYP6CY21*, and *CYP4CJ1* in *D. melanogaster* increases tolerance to cyantraniliprole [40]. In addition, the ThR and SR strains developed high-level cross-resistance to alpha-cypermethrin in previous studies [39,44]. The toxicity of alpha-cypermethrin to transgenic *Drosophila* was detected. *CSP1* and *CSP4* were overexpressed in *Drosophila* using the GAL4/UAS system to explore whether the susceptibility of the flies to thiamethoxam and spirotetramat changed after expressing the CSPs. The contact toxicities and gastric toxicities of thiamethoxam and alpha-cypermethrin were used in this study. Spirotetramat is mainly absorbed by the plant, transformed into spirotetramat-enol, and then transmitted to various parts of the plant to resist sucking pests [35]. Thus, we tested the susceptibility of *Drosophila* to spirotetramat by gastric toxicity bioassay. Transgenic flies showed higher resistance to thiamethoxam in terms of contact toxicities compared to gastric toxicities (Table 2 and Table 3). Results suggested that the defense effect of *CSP1* and *CSP4* against thiamethoxam is mainly concentrated in the process of insecticide-targeted transport. *CSPs* performed better when the gastric toxicity of alpha-cypermethrin was tested, indicating that *CSP1* and *CSP4* may play a defensive role after insects ingest pesticides (Table 2 and Table 3). However, the transgenic expression of *CSP4* significantly increased the resistance of *Drosophila* to alpha-cypermethrin in terms of contact toxicity, which showed that *CSP4* is also involved in preventing insecticide transport. Regarding the gastric toxicity of spirotetramat, the mortality of transgenic *Drosophila* was significantly reduced compared with that of the control except at concentrations of 5000 and 10,000 mg/L (Figure 3). Results showed that CSP4 confers tolerance to flies ingesting spirotetramat.

In summary, in this study of the resistance of cotton aphids to thiamethoxam, *CSP1* and *CSP4* were mainly expressed in body tissue. Fruit fly bioassay results also showed that they are mainly involved in the process of thiamethoxam penetrating the insect body wall. The overexpression of *CSP1* and *CSP4* is related to the production of resistance. In the gastric toxicity bioassay with spirotetramat, the expression of *CSP4* significantly increased fly tolerance, indicating that the overexpression of *CSP4* is associated with the susceptibility of cotton aphids to spirotetramat. Moreover, *CSP4* was mainly expressed in the body tissues. This evidence demonstrated that *CSP4* might also play other roles in SR strains, which requires further exploration. *CSP1* improved aphid resistance to alpha-cypermethrin in terms of gastric toxicities. *CSP4* can help *A. gossypii* endure exposure to alpha-cypermethrin in terms of both gastric toxicities and contact toxicities. Results showed that the overexpression of *CSP4* plays an important role in the resistance of aphids to alpha-cypermethrin. In summary, CSPs are related to *A. gossypii* resistance to several insecticides, and they may also have other functions.

## 4. Materials and Methods

### 4.1. Insects and Chemicals

Three strains of *A. gossypii* were used in this study. The susceptible (SS) strain was collected in Jilin province, China in 2008 [40,45]. The two other strains were resistant to thiamethoxam (ThR) and spirotetramat (SR) [40,45]. Resistant strains were established from the SS strain by consecutive selection with LC_30_ concentrations of thiamethoxam or spirotetramat. All aphids were reared on seedlings of *Gossypium hirsutum* (Linn.) in the laboratory at 22 ± 1 °C, 70 ± 10% relative humidity, and a photoperiod of 16:8 (L:D) h.

Thiamethoxam (25% WDG) and spirotetramat (Movento^®^, 24% SC) were purchased from Syngenta (Basel, Switzerland) and Bayer Crop Science (Monheim, Germany), respectively. Alpha-cypermethrin (98%) was supplied by Qingdao Hansen Biologic Science Co., Ltd. (Qingdao, China). Ex Taq DNA polymerase, DNA Marker DL2000, and PrimeScriptTM First-Strand cDNA Synthesis Kit with gDNA Eraser were purchased from TaKaRa (Kyoto, Japan). SGExcel FastSYBR Mixture (with ROX) was supplied by Sangon Biotech Co., Ltd. (Shanghai, China). The pGEM-T vector and the T7 RiboMAX™ Express RNAi System were purchased from Promega (Madison, WI, USA). The other technical reagents were of the highest purity available.

### 4.2. RNA Extraction, cDNA Synthesis and Gene Cloning

Total RNA of apterous adult aphids was extracted using RNAiso Plus (Takara, Kyoto, Japan) following the manufacturer’s protocol. To assess the quality of the RNA, we measured the RNA by a NanoPhotometer (IMPLEN, München, Germany) and performed in 1% agarose gel electrophoresis. cDNA was synthesized with PrimeScript^TM^ II Reverse Transcriptase (Takara, Kyoto, Japan) as the template for PCR, and the PrimeScript™ RT Reagent Kit with gDNA Eraser (Perfect Real Time) (Takara, Kyoto, Japan) was used for qPCR.

We filtered two upregulated CSP genes from previous transcriptomic studies [37]. *CSP1* (GenBank number: XM_027992041) was upregulated in the ThR strain, and *CSP4* (GenBank number: XM_027992042) was upregulated in the ThR and SR strains. Two open reading frames (ORFs) were amplified from cDNA using the primers listed in Table S1 of Supplementary data.

### 4.3. Quantitative PCR and Data Analysis

Quantitative PCR (qPCR) was performed using SGExcel FastSYBR Mixture (with ROX) with an ABI 7500 system (Applied Biosystems, Foster, USA). The primer sequences for qPCR (Table S1) were designed by Primer Premier 5.0 and synthesized by Sangon Biotech Co., Ltd. (Shanghai, China). The internal reference genes were glyceraldehyde-3-phosphate dehydrogenase (*GAPDH*) and elongation factor 1-alpha (*EF1α*) [46]. Each 20 μL reaction contained 10 μL of SGExcel FastSYBR Mixture, 0.4 μM of each primer and 1 μL of cDNA. The thermal cycling protocol was performed with the following conditions: 30 s at 95 °C, with 40 cycles of 5 s at 95 °C, followed by 34 s at 60 °C. The fluorescence signal was measured at the end of each extension step at 60 °C. After amplification, a dissociation step at 95 °C for 15 s, 60 °C for 1 min, and 95 °C for 15 s was performed to confirm that only specific products were amplified. Relative gene expression was analyzed by the 2^−ΔΔCT^ method [47]. Each treatment included three replicates. Significant differences were calculated using GraphPad InStat3 statistical software (GraphPad Software, 2000, San Diego, CA, USA).

### 4.4. DsRNA Synthesis and Diet-Mediated RNAi

DsRNAs of *ECFP*, *CSP1* and *CSP4* were synthesized using the T7 RiboMAX™ Express RNAi System (Promega, Madison, WI, USA) following the manufacturer’s instructions. The artificial diet and rearing methods used in this study were the same as those in previous reports [39,40]. We mixed dsRNA with the diet to a final concentration of 150 ng/μL, and dsRNA-*ECFP* was used as the control. Adults of the ThR/SR strain of *A. gossypii* were reared with the artificial diet for the experiment. After feeding the aphids for 48 h, we collected these aphids for qPCR to compute the efficiency of dsRNA knockdown of gene expression. To analyze the sensitivity of ThR strain aphids to thiamethoxam by knocking down *CSP1* and *CSP4*, and SR strain aphids to spirotetramat by knocking down *CSP4*, eighty aphids were fed a diet containing thiamethoxam (2 mg/L) or spirotetramat (2500 mg/L) with dsRNA-*CSP* or dsRNA-*ECFP* (150 ng/μL). Each treatment included three replicates. We recorded mortality after 48 h and corrected the mortality data using Abbott’s formula [48].

### 4.5. Construction of UAS-CSP Transgenic Drosophila and Bioassays

The pNP vectors [49] of tUAS-*CSP* were constructed, and the *CSP1* and *CSP4* coding sequences were inserted. Relevant primers are listed in Table S1 of Supplementary data. The UAS-*CSP* transformant lines were constructed by the *Drosophila* Center of Tsinghua University. The *D. melanogaster* strain carrying an attP40 docking site on chromosome 2 [y sc v nanos-integrase; attP40] was microinjected with recombinant vector. By balancing using standard techniques, the homozygous transformant line UAS-*CSP* was obtained.

We crossed virgin females or males of the Esg-GAL4 strain [y w; esg-Gal4 UAS-GFP/CyO] with UAS-*CSP* lines. The Esg-GAL4 strain drives gene expression in the midgut. The UAS-*CSP* males were crossed with virgin females of the Act5C-GAL4 strain [y w; act5C-Gal4/CyO] (drives gene expression in the whole body). The genotype of the cross of Esg > UAS-*CSP* F1 larvae expresses green fluorescent protein in the alimentary canal. Two cross-strain F1 adult offspring showed a straight-wing phenotype. The transformed UAS-*CSP* line crossed with [y sc v nanos-integrase; attP40] was used as the control.

The bioassay of Act5C > UAS-*CSP* F1 adult offspring was the topical application method [40,50]. The bioassay of Esg > UAS-*CSP* F1 adult offspring refers to a sucrose feeding method. Eight pairs of 2-day-old *Drosophila* adults were used in this study. Thiamethoxam and alpha-cypermethrin were dissolved in acetone, and the solutions were topical applied, then insects were fed with diet without any insecticides. The negative control was topical applied with acetone. The bioassay of Esg > UAS-CSP F1 adult offspring refers to a sucrose feeding method. Eight pairs of 2-day-old *Drosophila* adults were used in this study. We dissolved thiamethoxam and alpha-cypermethrin in 20% sucrose solution to 7 concentrations and spirotetramat to 3 concentrations; then, flies were fed with the solutions. A 20% sucrose solution without insecticide was used as a control. Mortality under alpha-cypermethrin was scored after 48 h, that under thiamethoxam was scored after 72 h, and that under spirotetramat was scored after 168 h. Each concentration was tested simultaneously in triplicate. *Drosophila* were reared under a photoperiod of 16:8 h (L:D) at 25 ± 1 °C. LC_50_ values were calculated via probit analysis using PoloPlus 2.0 (LeOra Software, Petaluma, USA).

## Figures and Tables

**Table 1:** *CSP1* and *CSP4* identified as significantly differentially transcribed between the ThR/SR and SS strains of *A. gossypii*.

	Gene	Transcriptome Data				qPCR Result	
		SS (FPKM)	ThR/SR (FPKM)	Log_2_ (FC)	FDR	Relative Expression Level	*p*-Value
ThR/SS	*CSP1*	15.22	24.77	0.71	<0.001	1.28	0.026
	*CSP4*	139.44	258.34	0.90	<0.001	2.30	<0.0001
SR/SS	*CSP4*	139.44	221.55	0.66	<0.001	6.08	0.0008

FC: fold change, FPKM of resistant/FPKM of susceptible samples. FPKM, fragments per kilobase of exon model per million mapped fragments. FPKM < 1 was a standard to judge the unigenes not expressed in one development stage. FDR ≤ 0.05 and the absolute value of Log2 (FC) ≥ 0 were used as thresholds to judge the significance of gene expression difference. Data taken from the transcriptome of *A. gossypi* [37].

**Table 2:** Log-dose probit-mortality data for thiamethoxam and α-cypermethrin against transgenic *Drosophila* broad tissue expressing *AgosCSP1* and *AgosCSP4*.

Insecticide	Gene	UAS-*CSPs* > [y sc v Nanos-Integrase; attP40] Strain					Act5C > UAS-*CSPs* Strain					
		LD_50_ (95% CL ^a^) (ng/per Adult)	Fit of Probit Line ^b^				LD_50_ (95% CL ^a^) (ng/per Adult)	Fit of Probit Line ^b^				RF at LD_50_^c^ (95% CL ^a^)
			Slope ± SE	*χ* ^2^	*p*	*df*		Slope ± SE	*χ^2^*	*p*	*df*	
Thiamethoxam	*CSP1*	22.01 (17.88–27.49)	2.80 ± 0.37	9.68	0.88	16	61.57 (49.39–81.17)	2.57 ± 0.40	9.68	0.88	16	2.80
	*CSP4*	13.06 (9.52–18.20)	1.74 ± 0.23	17.03	0.38	16	88.33 (61.24–157.61)	1.86 ± 0.30	19.72	0.23	16	6.76
α-cypermethrin	*CSP1*	0.29 (0.24–0.35)	3.00 ± 0.40	7.66	0.96	16	0.31 (0.25–0.38)	2.64 ± 0.36	11.87	0.75	16	1.06
	*CSP4*	0.12 (0.10–0.14)	3.32 ± 0.41	15.44	0.49	16	0.66 (0.52–0.91)	2.47 ± 0.41	6.89	0.98	16	5.76

^a^ Confidence limits. ^b^ Probit model fitted using POLO-PC (LeOra Software, 1987). ^c^ RF (resistance factors) = LD_50_ of Act5C > UAS-*CSP* strain /LD_50_ of UAS-*CSP* strain.

**Table 3:** Log-dose probit-mortality data for thiamethoxam and α-cypermethrin against transgenic *Drosophila* midgut expressing *AgoCSP1* and *AgoCSP4*.

Insecticide	Gene	UAS-*CSPs* > [y sc v Nanos-Integrase; attP40] Strain					Esg > UAS-*CSPs* Strain					
		LC_50_ (95% CL ^a^) (mg L^−1^)	Fit of Probit Line ^b^				LC_50_ (95% CL ^a^) (mg L^−1^)	Fit of Probit Line ^b^				RF at LC_50_^c^ (95% CL^a^)
			Slope ± SE	*χ* ^2^	*p*	*df*		Slope ± SE	*χ* ^2^	*p*	*df*	
Thiamethoxam	*CSP1*	5.36 (4.90–5.87)	8.94 ± 0.85	43.38	0.00	16	7.35 (7.00–7.85)	9.72 ± 1.39	7.56	0.96	16	1.37
	*CSP4*	6.27 (5.80–6.88)	6.99 ± 0.78	27.71	0.03	16	7.73 (7.18–8.75)	9.44 ± 1.42	26.07	0.05	16	1.23
α-cypermethrin	*CSP1*	1.49 (1.25–1.96)	3.45 ± 0.61	4.80	0.99	16	4.90 (4.11–5.89)	3.10 ± 0.34	17.30	0.37	16	3.29
	*CSP4*	1.03 (0.81–1.41)	2.27 ± 0.29	17.36	0.36	16	7.46 (6.04–9.72)	2.59 ± 0.32	17.87	0.33	16	7.27

^a^ Confidence limits. ^b^ Probit model fitted using POLO-PC (LeOra Software, 1987). ^c^ RF (resistance factors) = LC_50_ of Esg > UAS-*CSP* strain /LC_50_ of UAS-*CSP* strain.

## Data Availability

Data available when required to authors.

## Supplementary Materials

The following supporting information can be downloaded at: https://www.mdpi.com/article/10.3390/ijms23042356/s1.
