# Supplementary material for: Chemosensory Proteins Are Associated with Thiamethoxam and Spirotetramat Tolerance in Aphis gossypii Glover
Source: Int J Mol Sci. 2022 Feb 21;23(4):2356. doi: 10.3390/ijms23042356 (PMC8874399; doi:10.3390/ijms23042356)
Supplement: Supplementary file 1 [file ijms-23-02356-s001.zip › ijms-1571427-supplementary.pdf]

Table S1. Primers used in experiments.

Primer Name	Sequence (5'-3')	Application
GAPDH-F	AACAGTTTTTTGAGTGGCGGT	Real-Time PCR
GAPDH-R	TGGTGTCAACTTGGATGCGTA	Real-Time PCR
EF1a-F	CAGTCCCAGTAGGTCGTGTTG	Real-Time PCR
EF1a-R	TCAGTGGTGATGTTAGCAGGT	Real-Time PCR
CSP1-q-F	GAGAAACGTCGTCATATCCA	Real-Time PCR
CSP1-q-R	ATTGCATCTGGAACAATCCT	Real-Time PCR
CSP4-q-F	ATTGCAGTAGTCTGTGTTGT	Real-Time PCR
CSP4-q-R	CGGGTAAAATTTTTCTCAA	Real-Time PCR
CSP1-ORF-F	CGGTTGAACTATTCCATCGG	PCR
CSP1-ORF-R	TTATTGTATAACTAAATATTCCGCT	PCR
CSP4-ORF-F	ATGCCGATGATTATTGCTTAT	PCR
CSP4-ORF-R	GATAAATAGTGCATATAGGTATTGC	PCR
CSP1-ds-F	ggtgtaatacgactcactatagggACTCGATACGATTATATTG	dsRNA synthesis
CSP1-ds-R	ggtgtaatacgactcactatagggGTAGTATCGTTACTAAGT	dsRNA synthesis
CSP4-ds-F	ggtgtaatacgactcactatagggTTGGCTGCGTTCGCAGTG	dsRNA synthesis
CSP4-ds-R	ggtgtaatacgactcactatagggTTAGTAAAACCCTTTTTC	dsRNA synthesis
ECFP- dsRNA-F	ggtgtaatacgactcactataggTTACGCCAAGCTTGCATGCCT	dsRNA synthesis
ECFP- dsRNA-R	ggtgtaatacgactcactataggACTCCAGCAGGACCATGTGATC	dsRNA synthesis
CSP1-ORF-NheI-F	gctagcATGAATATTTTAACGATT	Make constructs
CSP1-ORF-XhoI-R	ctcgagCTAAATATTCCGCTTTTT	Make constructs
CSP1-ORF-BglII-R	agatctCTAAATATTCCGCTTTTT	Make constructs
CSP4-ORF-NheI-F	gctagcATGGATTCCAGAATTGCA	Make constructs
CSP4-ORF-XhoI-R	ctcgagTTAAAATTTAGTAAAACCC	Make constructs
CSP4-ORF-BglII-R	agatctTTAAAATTTAGTAAAACCC	Make constructs

dsRNA, double-stranded RNA; EF1a, elongation factor 1-alpha; F, forward; GAPDH, glyceraldehyde-3-phosphate dehydrogenase; ORF, open reading frame; R, reverse. Lower-case indicates the T7 RNA polymerase promoter. The restriction enzyme sequences are indicated by lowercase letters and underlined

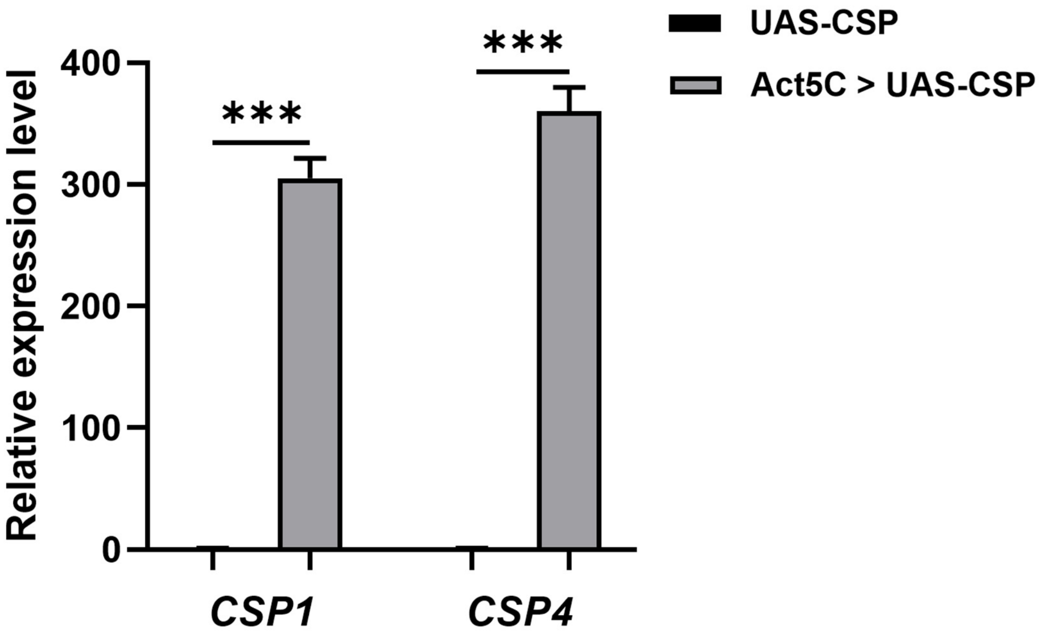

Figure S1. Transcription levels of *CSP1* and *CSP4* in the F1 straight wing adult genotype of Act5C > UAS- *CSPs* and UAS- *CSPs* > [y sc v nanos-integrase; attP40].

RpL32 and *ABP* were used as internal reference genes. Error bars indicate 95% confidence intervals (n = 3). *** Significant difference by Student's *t*-test ($p < 0.01$).

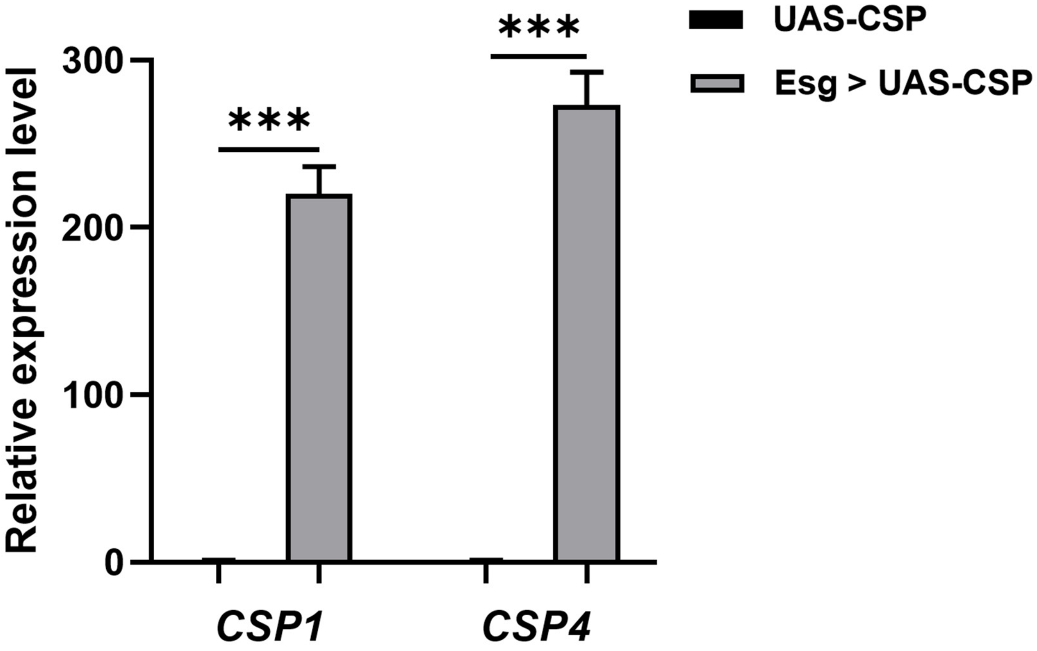

Figure S2. Transcription levels of *CSP1* and *CSP4* in the F1 straight wing adult genotype of Esg > UAS-*CSPs* and UAS-*CSPs* > [y sc v nanos-integrase; attP40]. *RpL32* and *ABP* were used as internal reference genes. Error bars indicate 95% confidence intervals (n = 3). *** Significant difference by Student's *t*-test ($p < 0.01$).

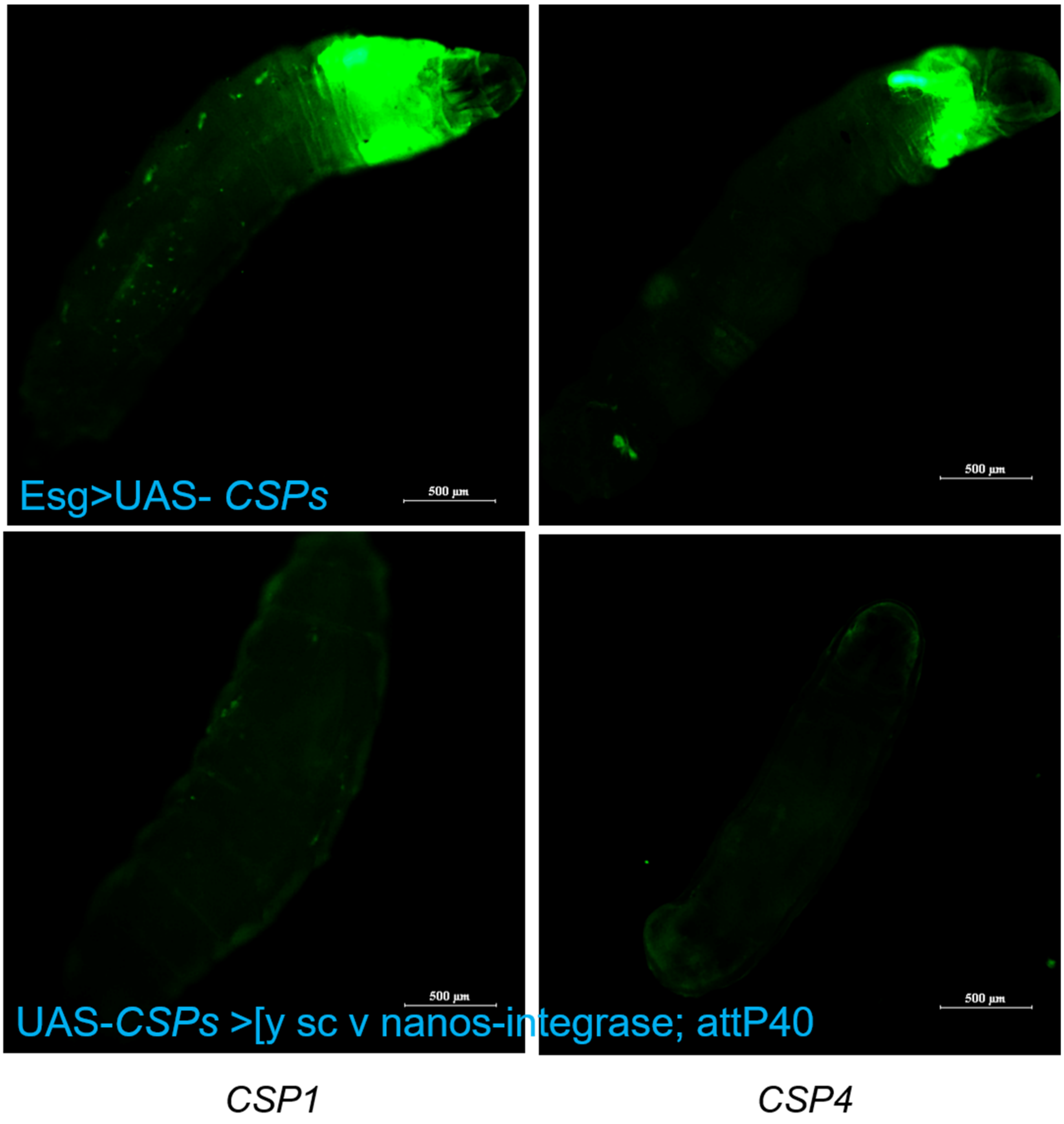

Figure S3. Fluorescence microscopy of F1 larval of Esg > UAS-*CSPs*.

The first line indicates F1 larval of Esg > UAS-*CSPs* strains; The second line demonstrate F1 larval of UAS-*CSPs* line crossing with [y sc v nanos-integrase; attP40].
